# Effects of heme oxygenase-1 recombinant *Lactococcus lactis* on the intestinal barrier of hemorrhagic shock rats

**DOI:** 10.1590/1414-431X20175601

**Published:** 2017-06-05

**Authors:** X.Y. Gao, X.F. Zhou, H. Wang, N. Lv, Y. Liu, J.R. Guo

**Affiliations:** 1 Department of Anesthesiology, Gongli Hospital, Second Military Medical University, Shanghai, China; 2 Shool of Medicine, Shandong University, Shandong, China

**Keywords:** Heme oxygenase-1, Hemorrhagic shock, Lactococcus lactis, Intestinal barrier, Intestinal inflammation

## Abstract

This study aimed to investigate the effects of heme oxygenase-1 recombinant *Lactococcus lactis* (LL-HO-1) on the intestinal barrier of rats with hemorrhagic shock. One hundred Sprague-Dawley male rats (280–320 g) were randomly divided into healthy control group (N group) and hemorrhagic shock group (H group). Each group was subdivided into HO1t, HO2t, HO3t, PBS and LL groups in which rats were intragastrically injected with LL-HO-1 once, twice and three times, PBS and *L. lactis* (LL), respectively. The mortality, intestinal myeloperoxidase (MPO) activity, intestinal contents of TNF-α, IL-10 and HO-1, and intestinal Chiu's score were determined. Results showed that in N group, the HO-1 content increased after LL-HO-1 treatment, and significant difference was observed in HO1t group and HO2t group (P<0.05). In H groups, MPO activity and Chiu's score decreased, but IL-10 content increased in LL-HO-1-treated groups when compared with PBS and LL groups (P<0.05). When compared with N group, the MPO activity reduced dramatically in LL-HO-1-treated groups. Thus, in healthy rats (N group), intragastrical LL-HO-1 treatment may increase the intestinal HO-1 expression, but has no influence on the intestinal barrier. In hemorrhagic shock rats, LL-HO-1 may significantly protect the intestinal barrier, and repeating the intragastrical LL-HO-1 treatments twice has the most obvious protection.

## Introduction

Hemorrhagic shock (HS) is a common complication of patients with severe trauma in clinical practice, and mortality is about 30–40% (1,2). In HS, the intestine is the first organ affected by the ischemia/reperfusion injury. Mucosal edema and villus rupture may be observed histopathologically. Bacteria from the intestines may enter blood circulation via the ruptured intestine (also known as bacterial translocation). The activities of macrophages and other immune cells increase, and the neutrophils are activated, resulting in damages to other organs and tissues (3–5). Heme oxygenase-1 (HO-1) is also known as heat shock protein 32 (HSP32) and is a member of heat shock protein (HSP) family. HO-1 is an important and unique inducible HO and can degrade the free heme released by aging or damaged red blood cells to produce carbon monoxide (CO), biliverdin (which will be further oxidized into bilirubin in the intestine) and divalent iron (Fe^2+^). HO-1 itself and its catalyst have anti-oxidative activities in the body. Evidence has confirmed that HO-1 is protective against HS via its anti-inflammation, anti-apoptosis, regulation of cell cycle, and maintenance of microcirculation (6–8).

In our lab, recombinant *Lactococcus lactis* expressing HO-1 (LL-HO-1) were successfully constructed by genetic engineering. Our previous study has confirmed that the bacteria may enter the ileum in healthy rats after intragastrical administration of LL-HO-1 at a certain dose. In the presence of HS and/or endotoxemia, HO-1 expression increased after LL-HO-1 treatment, which may protect the intestinal barrier via attenuating the intestinal inflammation (8–13 [Bibr B14]). However, whether the number of intragastrical LL-HO-1 treatments affect the protection of LL-HO-1 on the intestinal barrier is still unclear. In the present study, the intestinal barrier was evaluated in healthy rats and HS rats after repeated intragastrical LL-HO-1 treatments.

## Material and Methods

### Grouping and procedures

One hundred male Sprague-Dawley rats weighing 280–320 g were purchased from the Experimental Animal Center of Xuzhou Medical Collage. The protocol was approved by the Ethics Committee of Xuzhou Medical Collage, China. Rats were randomly assigned into healthy control group (N group, n=50) and hemorrhagic shock group (H group, n=50). Each group was further subdivided into 5 subgroups (n=10 per group): 1) one administration of LL-HO-1 (HO1t), 2) two administrations of LL-HO-1 (HO2t), 3) three administrations of LL-HO-1 (HO3t), 4) phosphate-buffered saline (PBS) group, and 5) *L. lactis* treatment group (LL). The time interval between treatments was 24 h, and 1 mL of the solution was administered intragastrically. In the HO1t, HO2t, HO3t, and LL groups, 2.5×10^9^ CFU/mL bacteria was administered. In PBS group, 1 mL of PBS was administered intragastrically. All animals received food deprivation before the experiment, but were given *ad libitum* access to food and water after.

At 24 h after the last intragastrical treatment, HS was induced in the H groups. In brief, rats were anesthetized and fixed in a supine position. Under an aseptic condition, the right femoral artery and the left femoral vein were separated. Bloodletting was done via the femoral artery, and the mean arterial pressure (MAP) was maintained at 35–40 mmHg for 60 min. Then, the collected blood and Ringer's lactate solution (1:2) were infused via the femoral vein within 30 min, and the blood pressure was maintained at ≥90% of that before the experiment, suggesting the successful resuscitation.

Animals were anesthetized with chloral hydrate at 1 h after HS in H group and at 24 h after the last intragastrical treatment in control group. Laparotomy was performed under an aseptic condition, and a 5-cm ileum was collected at the ileum terminal for further examinations. The mortality, intestinal myeloperoxidase (MPO) activity, and intestinal contents of TNF-α, IL-10 and HO-1 were determined. Colorimetry was used to determine the MPO activity (14), using a specific testing kit (Nanjing Jiancheng Bioengineering Institute, China) according to the manufacturer's protocols. The results of the MPO activity are reported as U/mg protein. In brief, 50 mg of small intestine tissue was weighed accurately and slurried with 950 µL medium. Reagent III (0.1 mL) was added to 0.9 mL homogenate and put in a 37°C water bath for 15 min. A 0.2 mL sample was added in the testing tube and in the control tube. The color-developing agent was added to the testing tube while distilled water was added to the control tube. Tubes were mixed and put in a 37°C water bath for 30 min. Then, reagent VII was added to each tube, mixed and put in a 37°C water bath of for 60 min. The absorbance level was measured immediately at 460 nm and 1 cm light path. MPO activity was measured with different absorbance levels. Immunohistochemistry followed by RichWin97 Software (Media Cybernetics, USA) were used to analyze the intestinal contents of TNF-α, IL-10 and HO-1. Ten areas of one field (*100) were chosen to calculate the gray value.

Histological examination was performed under a light microscope by experienced pathologists blind to the grouping in this study. Chiu's 6-point scoring system was employed to evaluate the intestinal mucosal injury, as follows: 0, normal; 1, enlargement of sub-epithelial space at the villus top; 2, moderate separation between epithelium and lamina propria; 3, significantly separated villi with destruction of the villus top; 4, destruction of the villus and exposure of capillaries in the lamina propria; 5, destruction, hemorrhage and ulcer in the lamina propria.

### Statistical analysis

Quantitative data are reported as means±SD. Intra-group comparisons were done with *t*-test, and intergroup comparisons with one-way analysis of variance. Statistical analysis was performed with SPSS version 19.5 (USA). A value of P<0.05 was considered to be statistically significant.

## Results

### Mortality

**Figure 1.**
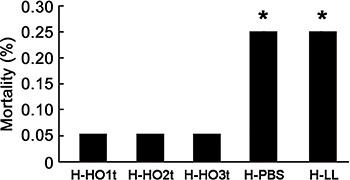
Mortality in hemorrhagic shock (H groups) treated once with *Lactococcus lactis* expressing heme oxygenase-1 (H-HO1t), twice (H-HO2t), three times (H-HO3t) or treated with phosphate-buffered saline (PBS) and *L. lactis* (LL). *P<0.05: compared to the remaining groups (ANOVA).

In H-HO1t, H-HO2t and H-HO3t groups, no animal died within 1 h after HS. However, in H-PBS and H-LL groups, 2 died before sample collection (mortality of 20%). In the control group, none died during the study. As shown in Figure 1, when compared with H-PBS and H-LL groups, the mortality was reduced significantly in the remaining groups (P<0.05).

### MPO activity of the intestine

**Figure 2.**
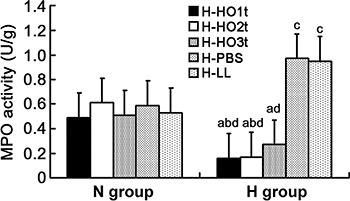
Myeloperoxidase (MPO) activity in normal (N) groups and hemorrhagic shock (H) groups treated with *Lactococcus lactis* expressing heme oxygenase-1 once (HO1t), twice (HO2t) or thrice (HO3t) or with phosphate-buffered saline (PBS) and *L. lactis* (LL). ^a^P<0.05, compared with H-PBS and H-LL; ^b^P<0.05, compared with H-HO3t; ^c^P<0.05, compared with N; ^d^P<0.05, compared with N group (ANOVA). Data are reported as means±SD.

As shown in Figure 2, the intestinal MPO activity in H-PBS and H-LL groups increased dramatically when compared with N-PBS and N-LL groups (P<0.05). MPO activity in H-HO1t, H-HO2t and H-HO3t groups reduced significantly when compared with H-LL and H-PBS groups (P<0.05). MPO activity in H-HO1t and H-HO2t groups reduced significantly when compared with H-HO3t group (P<0.05). MPO activity in H-HO1t, H-HO2t and H-HO3t groups reduced significantly when compared with N-HO1t, N-HO2t and N-HO3t groups, respectively (P<0.05).

### Contents of TNF-α, IL-10 and HO-1 in the intestines

**Figure 3.**
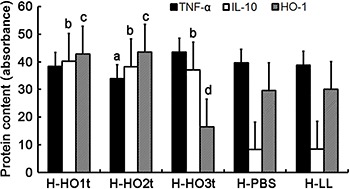
Levels of TNF-α, IL-10 and heme oxygenase-1 (HO-1) in the intestine of hemorrhagic shock (HS) rat groups treated with *Lactococcus lactis* expressing heme oxygenase-1 once (HO1t), twice (HO2t) or thrice (HO3t) or with phosphate-buffered saline (PBS) and *L. lactis* (LL). ^abc^P<0.05: compared with the corresponding PBS and LL groups (ANOVA). Data are reported as means±SD.

As shown in Figure 3, when compared with H-PBS and H-LL groups, the intestinal IL-10 content in H-HO1t, H-HO2t and H-HO3t groups increased significantly, TNF-α content in H-HO2t group reduced, HO-1 content in H-HO1t and H-HO2t groups increased, but H-HO-1 content reduced significantly in H-HO3t group (P<0.05).

### HO-1 content in control groups

**Figure 4.**
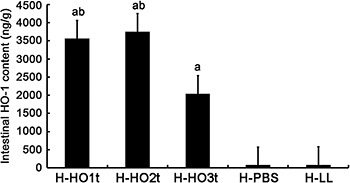
Content of heme oxygenase-1 (HO-1) in the intestine of different N groups treated with *Lactococcus lactis* expressing heme oxygenase-1 once (HO1t), twice (HO2t) or thrice (HO3t) or with phosphate-buffered saline (PBS) and *L. lactis* (LL) (ng/g). ^a^P<0.05: compared with PBS and LL groups; ^b^P<0.05: compared with HO3t group (ANOVA). Data are reported as means±SD.

As shown in Figure 4, when compared with N-PBS and N-LL groups, the HO-1 content in N-HO1t, N-HO2t and N-HO3t groups increased, and a significant difference was observed between N-HO1t group and N-HO2t group (P<0.05).

### Chiu's scores

**Figure 5.**
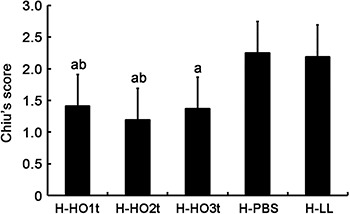
Chiu's score of hemorrhagic shock (H) rats treated with *Lactococcus lactis* expressing heme oxygenase-1 once (HO1t), twice (HO2t) or thrice (HO3t) or with phosphate-buffered saline (PBS) and *L. lactis* (LL). ^a^P<0.05: compared with PBS and LL; ^b^P<0.05: compared with HO1t and H-HO3t (ANOVA). Data are reported as means±SD.

**Figure 6.**
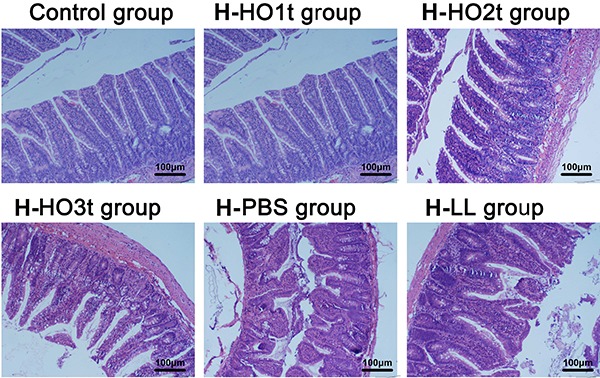
Pathological examination of intestinal tissue of hemorrhagic shock (H) groups treated with *Lactococcus lactis* expressing heme oxygenase-1 once (HO1t), twice (HO2t) or thrice (HO3t) or with phosphate-buffered saline (PBS) and *L. lactis* (LL). Control group refers to healthy rats (HE staining; ×100).

As shown in Figures 5 and 6, the morphology of the intestine was normal, and further comparisons were not performed in the N groups. When compared with H-PBS and H-LL groups, the Chiu's score reduced significantly in H-HO1t, H-HO2t and H-HO3t groups; when compared with H-HO1t and H-HO3t groups, the Chiu's score reduced significantly (P<0.05) in H-HO2t group.

## Discussion

A large number of studies confirm that the intestinal barrier is the first to be damaged following HS and other situations of low blood perfusion, presenting pathological changes (4). The ischemia/reperfusion and neutrophil (PMN) activation due to HS may cause the release of pre-inflammatory cytokines and the production of a variety of reactive oxygen species, which may lead to systemic inflammatory response syndrome, resulting in organ dysfunction and damage to cells and tissues (15,16). Under normal conditions, intestinal epithelial cells form a potent barrier via intercellular connections and adhesion, which protect the intestine against injury (17). In the presence of inflammation, a large amount of activated PMNs may exert mechanical effects via their pseudopodia and release reactive oxygen species, causing damage to the intestinal barrier, mucosal edema and intestinal barrier dysfunction, which is an important mechanism of intestinal inflammation. Activated PMN may release MPO, which activity reflects the number of PMN (18). Chiu's score, which is a morphological parameter reflecting the intestinal function (19), was used to evaluate the intestinal mucosal injury. The intestine is a major TNF-α-secreting organ and a major source of TNF-α in HS (20). IL-10 is the most important anti-inflammatory cytokine in the intestinal immune system. IL-10 may inhibit immune function and down-regulate inflammation, and thus has been a key maker of anti-inflammation in the intestine (21). The anti-inflammatory effect of IL-10 is mediated by HO-1, and one may induce the expression of the other, forming a positive feedback in the anti-inflammatory process (22). Regulating the secretion of the HO family members (HO-1, HO-2 and HO-3) is a promising way of protecting against injury. For example, inducing the expression of free HO-1 exerts anti-oxidative effects and protects the organs against damage (23). Thus, HO-1 has become an important anti-oxidant in human body (8,24,25).

In the present study, the MPO activity and Chiu's scores in control groups were comparable, suggesting that intragastric LL-HO-1 treatment has no influence on the immune function of the intestine and does not disrupt the intestinal barrier. Thus, the TNF-α and IL-10 contents were not measured in control groups. In H-PBS and H-LL groups, the MPO activity and Chiu's scores were significantly higher than in control group, which is related to the compromised intestinal barrier and intestinal inflammation following stress. MPO activity in H-HO1t, H-HO2t and H-HO3t groups was markedly lower than in control groups, which is associated with HO-1-induced reduction in intestinal stress (decrease in PMN activation and inhibition of intestinal inflammation). However, when compared with H-HO1t and H-HO3t groups, the protective effects were better in H-HO2t group, suggesting the improvement of intestinal inflammation and better intestinal barrier.

The TNF-α content was comparable in H groups, indicating similar stress levels. However, the contents of IL-10 and HO-1 in H-HO1t, H-HO2t and H-HO3t groups were significantly higher than in control group, suggesting that the anti-inflammatory cytokine increases, which is helpful for the anti-inflammatory effects in late stages. In this study, the inflammatory cytokine was assessed only within 1 h after HS, which was one of limitations of this study.

In this study, LL-HO-1-treated rats survived after intragastrical treatment, leading to the increased HO-1 expression in the intestine, but the HO-1 expression did not increase with an increased number of intragastrical treatments. On the contrary, the HO-1 expression reduced in the intestine after two intragastrical treatments, which was consistent with the findings in the H group. Compared to the control and HS groups, the HO-1 expression increased significantly and the intestinal barrier was better after two LL-HO-1 treatments. This may be explained as follows: Firstly, the lactobacillus expression system used in our study is an international, food-grade NICE system widely used for the construction of *L. lactis* expressing exogenous genes (26). However, the release of target proteins by the recombinant *L. lactis* is relatively limited, and the bacteria cannot sustain releasing the target protein in the intestine. Once the *L. lactis* activity reduces, the synthesis and release of its target protein is also reduced (27,28). Secondly, there is evidence that the HO-1 protection is dependent on the HO-1 expression, and excess HO-1 expression may promote lactate dehydrogenase release and reduce glutathione S-transferase, leading to the disruption of cell integrity (29).

Taken together, intragastrical treatment with LL-HO-1 may induce HO-1 expression in the intestine of healthy rats, and protect the intestinal barrier against HS-induced stress. However, whether there is a threshold of HO-1 expression in the HO-1-induced intestinal protection and whether this treatment is related to the activation of endogenous HO-1 are still unclear and require further study.

In conclusion, intragastrical LL-HO-1 treatment may induce HO-1 expression in the intestine without affecting the intestinal barrier in healthy rats. In HS rats, intragastrical LL-HO-1 treatments, especially twice, can significantly protect the intestinal barrier.
